# Association between the rs6313 polymorphism in the *5-HTR2A* gene and the efficacy of antipsychotic drugs

**DOI:** 10.1186/s12888-023-05165-1

**Published:** 2023-09-19

**Authors:** Yulong Wang, Xingru Tan, Zhoufangyuan Chen, Bide Zhang, Yunzhi Gao, Yanlong Wang

**Affiliations:** 1 Lin He’s Academician Workstation of New Medicine and Clinical Translation in Jining Medical University, Jining Medical University, Jining, China; 2 College of Teacher Education, Qilu Normal University, Jinan, China; 3 College of Basic Medicine, Jining Medical University, Jining, China

**Keywords:** Genetic polymorphism, Schizophrenia, *5-HTR2A*, rs6313, Antipsychotic drugs

## Abstract

**Background:**

Prescribing the optimal antipsychotic treatment to schizophrenia is very important as it is well established that patients have different sensitivity to the available antipsychotic drugs. The genotype of the *HTR2A* T102C (rs6313) polymorphism has been suggested to affect the efficacy of antipsychotic drugs, but the results of different studies have been inconsistent

**Methods:**

In this study, a meta-analysis was used to ascertain the association between allele and genotype polymorphism of rs6313 and the efficacy of antipsychotic drugs. Related studies publicated from January 1995 to December 2021 were retrieved from PubMed, Embase, ScienceDirect, and Web of Science databases. The correlations between allele and genotype polymorphism of rs6313 and the responder rate and scale score reduction rate of antipsychotics were analyzed. In addition, subgroup analyses were performed on time, drug, and ethnicity.

**Results:**

A total of 18 studies were included. The meta-analysis showed that allele and genotype polymorphisms at the rs6313 locus overall were not associated with antipsychotic drug responder rate or scale score reduction rate. Ethnicity subgroup analysis showed that antipsychotic drugs were more effective in patients with allele T in the Caucasian population. Indian patients with the TT genotype had the lowest scale score reduction rate and poor drug treatment effect. East Asian patients with the TC genotype had better treatment effect, whereas in patients with the CC genotype, the treatment was less effective. Drug subgroup analysis showed that patients with the TC genotype treated with clozapine had the highest responder rate and score reduction rate.

**Conclusions:**

The association between rs6313 polymorphism and the efficacy of antipsychotic drugs is mainly influenced by drug and ethnicity. Caucasian patients with the T allele respond better to drug therapy, and Asian patients with TC genotype. The TC genotype was also a good predictor of the efficacy of clozapine treatment.

## Background

Schizophrenia is a common severe mental disease that affects many young adults and has a prolonged course [1]. Olanzapine, clozapine, risperidone, and other atypical antipsychotics have been widely used in the clinical treatment of schizophrenia [2]. Genetic polymorphisms can lead to differences in antipsychotic efficacy among individuals. Serotonin 2A receptor (*5-HTR2A*) gene polymorphisms, particularly polymorphism rs6313, have been suggested to be associated with the efficacy of antipsychotic drugs [3–5]. Polymorphism rs6313 is located in codon 102 of *5-HTR2A* exon 1 and allele T is replaced by C.

It has been found that the Scale for Assessment of Negative Symptoms (SANS) score reduction rate in individuals with rs6313 TT genotype was higher than that in carriers of other genotypes [6]. Further, risperidone treatment was more effective in patients with severe schizophrenia that had the CC genotype [7]. Alladi et al. [8] and Yan et al. [9] suggested that genetic variation of rs6313 was not associated with the pharmacodynamic response to risperidone. In some studies, clozapine was found to be not associated with the pharmacodynamic response in carriers of the C allele in the Asian populations [10], whereas in the Caucasian population, this drug had stronger effect in the carriers of the T allele [11]. Maffioletti et al. found that efficacies of risperidone and olanzapine were not associated with rs6313 variation, when analysed separately by comparing responders and non-responders in early drug therapy. However, considering the two drugs together, it was observed that carriers of the T allele had a higher responder rate than non-carriers [12]. Therefore, current data regarding the association between the rs6313 polymorphism in the *5-HTR2A* gene and efficacy of antipsychotic drugs are inconsistent.

This discrepancy may be caused by the differences in ethnicity, time, sample size, and drugs analysed [13, 14]. In addition, the published studies used different methods to evaluate antipsychotic drug efficacy and gene polymorphism. Evaluation methods of antipsychotic drug efficacy mainly include responder rate and scale score reduction rate [8]. The responder rate is the proportion of samples whose scale score reduction rate exceeds the threshold of the total samples. Gene polymorphisms were mainly characterised as allele polymorphism and genotype polymorphism. In this study, we performed a meta-analysis of the association between the rs6313 polymorphism in the *5-HTR2A* gene and the responder rate and scale score reduction rate of antipsychotic drugs in the past 25 years. We hope that our results regarding the association between the rs6313 polymorphism and antipsychotic drug efficacy provide a molecular genetic basis for the improved treatment of patients with schizophrenia.

## Methods

### Data retrieval strategy

This study was guided by the standard PRISMA protocol and has been registered in PROSPERO (registration number: CRD42022309940). All studies that examined the relationship between *5-HTR2A* gene polymorphisms and antipsychotic drug efficacy and were published in English literature were retrieved from PubMed, Embase, ScienceDirect, and Web of Science databases. The publication period was from January 1995 to December 2021. The search terms included: “Schizophrenia”, “5-hydroxytryptamine 2A receptor”, “HTR2A”, “5-HT2a”, “5HT2A”, “5-HTR2A”, “5-HT_(2A)”, “5-HT2AR”, “single nucleotide polymorphism”, “polymorphism”, and “SNP”. Two authors (Xingru Tan and Zhoufangyuan Chen) independently performed a systematic review using the same criteria and resolved any inconsistency through a discussion with another author (Bide Zhang) to make the final decision.

### Inclusion and exclusion criteria

According to the method of Li et al. [15], the included studies had to meet the following criteria: a) the original data were in published studies that assessed the relationship between the rs6313 polymorphism of the *5-HTR2A* gene and antipsychotic drug efficacy; b) the subjects of the original study were patients with schizophrenia and other psychotic disorders or affective disorders; c) during the experiment, all subjects took antipsychotic drugs orally, and no longer received any other antipsychotic treatment; d) in the original study, drug efficacy was evaluated by the Positive and negative symptom scale (PANSS), Brief psychiatric rating scale (BPRS), Clinical global impression (CGI), Global assessment scale (GAS), Scale for the assessment of negative symptoms (SANS), and prior criteria, and the reduction rate was evaluated or patients were divided into responders and non-responders according to the reduction rate; e) the statistical methods of the original study were appropriate, and there were clear genotype or allelic frequency data. Referring to the method of Yang et al. [16], a study was excluded if: a) it was a review, a conference abstract, a commentary, a news reports or other similar type of publication; b) it was a repeated publications; c) it had incomplete or insufficient data; d) it was irrelevant.

### Data extraction

Two evaluators (Xingru Tan and Yunzhi Gao) separately extracted the data from the included study and resolved any inconsistency through a discussion with another author (Yulong Wang) to make the final decision. Extracted data included: first author, year of publication, sample size, country of origin, ethnicity, age, patient attributes, diagnostic criteria, drugs taken, follow-up period, evaluation scale, reduction rate, criteria for defining a responder, and number of patients who were responders and non-responders stratified by different genotypes.

### Statistical analysis

Review Manager 5.3 software was used for data processing and meta-analysis. Heterogeneity was assessed using the Cochran’s Q test and I^2^ statistic. When *P* ≤ 0.10 and I^2^ > 50%, it indicates heterogeneity among different studies, and the random effect model was used to merge the data. When *P* > 0.10 and I^2^ ≤ 50%, it indicates no heterogeneity between studies. Then, the odds ratio (OR) value of the responder rate, standardized mean difference (SMD) value of the scale score reduction rate, and 95% confidence interval were calculated after the combination of the data extracted from eligible studies. Differences were considered statistically significant when *P* ≤ 0.05. The funnel plot was used to assess publication bias. The sensitivity analysis was carried out by one-by-one elimination method [17].

## Results

### Eligible studies

**Fig. 1:**
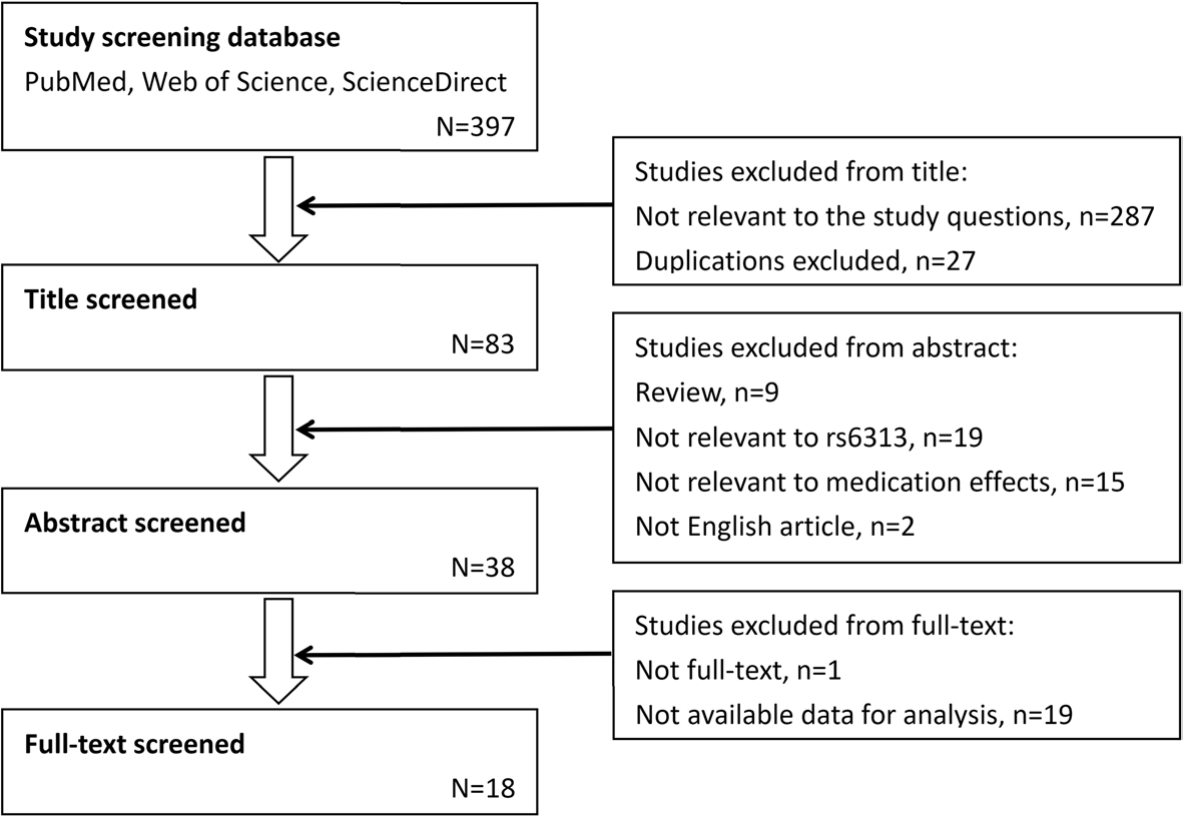
Flowchart of the study selection process

As shown in Fig. 1, 397 studies were initially retrieved from the databases. Next, we analysed the papers and excluded irrelevant and duplicate studies (314 studies in total). Having read abstracts, we exclude reviews, studies that were not about the rs6313 polymorphism, non-pharmacodynamics studies, and articles that were published in languages other than English (45 studies in total). Next, we read full texts of the remaining papers and removed studies with unavailable full text or lacking data (20 studies in total). Finally, the remaining 18 studies were used for meta-analysis.

### Characteristics of analysed studies

A total of 2,838 patients were included in the eligible studies (Table 1). Antipsychotics included risperidone, olanzapine, clozapine, and haloperidol. In addition, the study of Maffioletti et al. [12] divided Italian Caucasian population into two groups, treated with risperidone and olanzapine, respectively, and analysed them separately. The study of Yan et al. [9] divided East Asian population in China into two groups, Shanghai and Henan, and analysed them separately. Therefore, in the subsequent meta-analysis, the two sets of data from the above two studies were separated for statistical treatment. Data extraction revealed that 13 studies analysed the responder rate of different drug treatments (Table 2). The criteria for defining responders mainly included BPRS reduction ≥ 20%, total PANSS reduction ≥ 30%, and total PANSS reduction ≥ 50%. Seven studies analysed the scale score reduction rate of different drug treatments. The scale score reduction rate mainly included the reduction of PANSS score, reduction of total PANSS score, and reduction rate of total PANSS score (Table 3).

**Table 1:** General information of the included literatures

No	First author	Year of Publication	Cases (n)	Country of origin	Ethnicity	Age (year)	Patient attributes	Diagnostic criteria	Drug	Follow-up period (weeks)
1	Arranz et al. [11]	1995	149	USA	Caucasian	—	Schizophrenia	DSM-III-R	Clozapine	≥ 12
2	Malhotra et al. [18]	1996	70	USA	—	30–44	Schizophrenia or schizoaffective disorder	DSM-III-R	Clozapine	10
3	Masellis et al. [19]	1998	181	USA	Mixture	24–42	Schizophrenia	DSM-III-R	Clozapine	≥ 24
4	Lin et al. [10]	1999	97	China	East Asian	34–41	Schizophrenic disorders	DSM-IV	Clozapine	≥ 8
5	Joober et al. [20]	1999	102	Canada	Caucasian	31–52	Schizophrenia	DSM-IV	Clozapine	_
6	Ellingrod et al. [6]	2002	41	USA	Caucasian	18–65	Schizophrenia	DSM-IV, BPRS 9	Olanzapine	6
7	Mata-Pastor et al. [21]	2002	51	UK	Caucasian	18–73	Schizophrenia	DSM-IV	Olanzapine	≥ 12
8	Anttila et al. [22]	2007	94	Finland	Caucasian	34–60	Schizophrenia	DSM-IV	main Clozapine	4
9	Kim et al. [23]	2008	100	Korea	East Asian	21–41	Schizophrenia	DSM-IV	Risperidone	4
10	Thomas et al. [24]	2008	115	India	Indian	18–65	Schizophrenia or schizoaffective disorder	DSM-IV	Olanzapine	6
11	Olajossy-Hilkesberger et al. [25]	2011	95	Australia	Caucasian	17–68	Paranoid schizophrenia	DSM-IV	Olanzapine	≥ 6
12	Yan et al. (Shanghai) [9]	2015	111	China	East Asian	18–60	Schizophrenia	DSM-IV	Risperidone	4
	Yan et al. (Henan) [9]	2015	87	China	East Asian	16–55	Schizophrenia	DSM-IV	Risperidone	4
13	Gareeva et al. [26]	2015	97	Russia	Russian	15–54	Paranoid schizophrenia	ICD-10	Haloperidol	3
14	Kaur et al. [27]	2017	331	India	Indian	18–55	Schizophrenia	ICD-10 DCR	Risperidone	12
15	Shi et al. [28]	2017	546	China	East Asian	19–51	Schizophrenia	DSM-IV	Risperidone	4
16	Alladi et al. [8]	2019	109	India	Indian	21–46	Schizophrenia		Risperidone	4
17	Yan et al. [29]	2020	241	China	East Asian	18–60	Schizophrenia	ICD-10	Olanzapine	4
18	Maffioletti et al. (risperidone) [12]	2020	121	Italy	Caucasian	28–54	Schizophrenia	DSM-IV	Risperidone	2
	Maffioletti et al. (olanzapine) [12]	2020	100	Italy	Caucasian	24–48	Schizophrenia	DSM-IV	Olanzapine	2

*BPRS* Brief psychiatric rating scale, *DSM* The diagnostic and statistical manual of mental disorders, *ICD* International classification of diseases, *ICD-10 DCR* International classification of diseases-10 diagnostic criteria for research

**Table 2:** Responder rate of the included literatures

Study	Evaluation scale	Define responder	Allele				Genotype					
			T		C		TT		TC		CC	
			R	NR	R	NR	R	NR	R	NR	R	NR
Arranz et al. 1995 [11]	GAS	GAS improvement > 20	84	36	100	78	15	9	54	18	23	30
Malhotra et al. 1996 [18]	BPRS	BPRS reduction ≥ 20%	18	45	24	53	2	9	14	27	5	13
Masellis et al. 1998 [19]	BPRS	BPRS reduction ≥ 20%	86	79	104	93	19	18	48	43	28	25
Joober et al. 1999 [20]	Priori criteria	_	31	38	47	88	7	7	17	24	15	32
Mata-Pastor et al. 2002 [21]	PANSS,GAS	GAS improvement > 20	24	22	32	24	6	3	12	16	10	4
Anttila et al. 2007 [22]	GGI	Hospital and mental health care records and personal interview	29	27	57	73	3	4	23	19	17	27
Kim et al. 2008 [23]	GGI-I	GGI score < 4	74	22	92	12	13	6	48	10	22	1
Thomas et al. 2008 [24]	PANSS	PANSS reduction ≥ 30%	75	61	59	35	17	15	41	31	9	2
Olajossy-Hilkesberger et al. 2011 [25]	PANSS	Total PANSS reduction ≥ 30%	39	47	49	55	8	6	23	35	13	10
Kaur et al. 2017 [27]	PANSS	PANSS reduction rate ≥ 50%	182	108	218	154	37	19	108	70	55	42
Shi et al. 2017 [28]	PANSS	PANSS reduction ≥ 50%	386	230	308	168	110	66	166	98	71	35
Alladi et al. 2019 [8]	PANSS	PANSS reduction≧20%	71	19	97	31	12	4	47	11	25	10
Yan et al. 2020 [29]	PANSS	PANSS reduction rate ≥ 50%	173	117	117	75	51	39	71	39	23	18

*BPRS* Brief psychiatric rating scale, *GAS* Global assessment scale, *GGI* Clinical global impression, *NR* non-responders, *PANSS* Positive and negative symptom scale, *R* responders

**Table 3:** Scale score reduction rate of the included literatures

Study	Cohort	Evaluation scale	Therapy efficiency	TT		TC		CC	
				**Mean**	**SD**	**Mean**	**SD**	**Mean**	**SD**
Lin et al. 1999 [10]	-	BPRS	BPRS total score change	8.7	1.2	9.5	1.1	8.6	1.9
Ellingrod et al. 2002 [6]	-	SANS	Reduction rate of SANS score	46	12	20	6	20	6
Olajossy-Hilkesberger et al. 2011 [25]	-	PANSS	Reduction of total PANSS score	28.7	20.9	22.3	18.8	33.4	18.5
Yan et al. 2015 [9]	Shanghai	PANSS	Reduction of total PANSS score	24.98	15.47	24.73	13.62	19.72	10.96
	Henan	PANSS	Reduction of total PANSS score	17	16.08	20.21	17.97	19.94	15.05
Gareeva et al. 2015 [26]	-	PANSS	Reduction rate of PANSS total score	23.3	6.25	23.19	6.38	23.17	6.36
Alladi et al. 2019 [8]	-	PANSS	Reduction of PANSS score	27.06	18.3	35.8	19.8	32.62	20.6
Maffioletti et al. 2020 [12]	Risperidone	PANSS	Reduction rate of total PANSS score	27.1	11	21.7	10.9	23.2	12.7
	Olanzapine	PANSS	Reduction rate of total PANSS score	18.9	17.6	24.3	11.3	16.2	14.7

*BPRS* Brief psychiatric rating scale, *PANSS* Positive and negative symptom scale, *SANS* Scale for the assessment of negative symptoms

### Meta-analysis

#### Meta-analysis of the relationship between rs6313 polymorphism and responder rate

A total of 2,184 patients with schizophrenia were included. Allelic variation (T > C polymorphism) and genotype variation (TT > TC, TT > CC, and TC > CC polymorphism) were both meta-analysed for the association with pharmacodynamics. However, differences in time, drug, and ethnicity may lead to differences in the results of association analyses [13, 14]. Therefore, subgroup analyses were further performed using time (1995–2000, 2001–2010, or 2011–2021), drug (risperidone, olanzapine, or clozapine), and ethnicity (East Asian, Caucasian, Indian, or others) as factors (Table 4).

**Table 4:** Meta-analysis of the relationship between antipsychotic drug responder rate and rs6313 T > C, TT > CC, TT > TC, and TC > CC polymorphism

Polymorphism	Total		Time					
			**1995–2000**		**2001–2010**		**2011–2021**	
	**OR[95%CI]**	***P* value**	**OR[95%CI]**	***P* value**	**OR[95%CI]**	***P* value**	**OR[95%CI]**	***P* value**
**T/C**	1.02[0.90,1.16]	0.72	1.25[0.97,1.62]	0.09	0.80[0.58,1.11]	0.18	1.00[0.86,1.18]	0.96
**TT/TC**	0.93[0.74,1.16]	0.50	0.81[0.49,1.35]	0.42	0.84[0.47,1.50]	0.56	0.98[0.75,1.29]	0.91
**TT/CC**	1.00[0.77,1.30]	0.99	1.36[0.80,2.31]	0.26	0.42[0.18,0.97]	0.04	1.03[0.74,1.43]	0.87
**TC/CC**	1.12[0.78,1.59]	0.54	1.70[0.88,3.29]	0.11	0.52[0.15,1.75]	0.29	1.04[0.75,1.43]	0.83

Polymorphism	Ethnicity								Medicine					
	**East Asian**		**Caucasian**		**Indian**		**Else**		**Risperidone**		**Olanzapine**		**Clozapine**	
	**OR[95%CI]**	***P* value**	**OR[95%CI]**	***P* value**	**OR[95%CI]**	***P* value**	**OR[95%CI]**	***P* value**	**OR[95%CI]**	***P* value**	**OR[95%CI]**	***P* value**	**OR[95%CI]**	***P* value**
**T/C**	0.88[0.72,1.07]	0.20	1.32[1.02,1.72]	0.04	1.07[0.83,1.37]	0.59	0.95[0.66,1.36]	0.78	0.98[0.82,1.17]	0.80	0.88[0.68,1.13]	0.31	1.27[1.00,1.61]	0.05
**TT/TC**	0.85[0.62,1.16]	0.31	1.13[0.65,1.95]	0.67	1.04[0.65,1.66]	0.87	0.81[0.41,1.60]	0.54	0.98[0.72,1.33]	0.88	0.95[0.63,1.44]	0.81	0.79[0.49,1.29]	0.34
**TT/CC**	0.79[0.53,1.18]	0.25	1.56[0.88,2.79]	0.13	1.11[0.64,1.94]	0.70	0.86[0.40,1.85]	0.71	0.93[0.64,1.35]	0.69	0.82[0.47,1.44]	0.49	13.4[0.81,2.22]	0.26
**TC/CC**	0.92[0.50,1.70]	0.80	1.22[0.52,2.86]	0.64	1.07[0.53,2.15]	0.85	1.07[0.59,1.94]	0.82	1.01[0.67,1.54]	0.95	0.59[0.26,1.35]	0.21	1.74[1.05,2.91]	0.03

*CI* confidence interval, *OR* odds ratio value of the responder rate. Differences were considered statistically significant when *P* ≤ 0.05

Meta-analysis showed that allelic variation and genotype variation were not associated with drug efficacy (*P* value of T > C, TT > TC, TT > CC, and TC > CC were 0.72, 0.50, 0.99, and 0.54, respectively). For the allelic variation, subgroup analyses showed that the T > C polymorphism was associated with drug efficacy in the Caucasian population (ethnicity subgroup analysis, *P* = 0.04) and clozapine-treated population (drug subgroup analysis, *P* = 0.05): drug efficacy was higher in the carriers of the T allele in both groups (OR value of Caucasian population and clozapine-treated population were 1.32 and 1.27, respectively). With regards to the genotype variation, the TT > CC genotype polymorphism in the 2001–2010 population (time subgroup analysis) was associated with drug efficacy, which was higher in the patients with CC genotype (OR = 0.42). Further, the TC > CC genotype polymorphism in clozapine-treated population (drug subgroup analysis) was associated with drug efficacy, and the drug was effective in the patients with the TC genotype (OR = 1.74).

#### Meta-analysis of the relationship between the rs6313 polymorphism and scale score reduction rate

Nine studies from seven papers analysed the association between the rs6313 polymorphism and scale score reduction rate. A total of 858 patients with schizophrenia were included. Meta-analysis was performed on the TT > TC, TT > CC, and TC > CC polymorphisms. Subgroup analyses were further performed using time (1995–2000, 2001–2010, or 2011–2021), drug (risperidone, olanzapine, clozapine, or haloperidol), and ethnicity (East Asian, Caucasian, Indian, or Russian) as factors (Table 5). The combined results showed that there was no association between the three genotype variations and scale score reduction rate.

**Table 5:** Meta-analysis of the relationship between antipsychotic drug scale score reduction rate and rs6313 TT > CC, TT > TC, and TC > CC polymorphism

Polymorphism	Total		Time					
			**1995–2000**		**2001–2010**		**2011–2021**	
	**SMD[95%CI]**	***P* value**	**SMD[95%CI]**	***P* value**	**SMD[95%CI]**	***P* value**	**SMD[95%CI]**	***P* value**
**TT/TC**	0.16[-0.24,0.56]	0.44	-0.69[-0.98,-0.40]	0.00	2.71[2.11,3.32]	0.00	-0.02[-0.28,0.24]	0.87
**TT/CC**	0.27[-0.07,0.61]	0.12	0.06[-0.22,0.34]	0.66	2.71[2.11,3.32]	0.00	0.03[-0.18,0.24]	0.77
**TC/CC**	0.12[-0.13,0.37]	0.35	0.58[0.29,0.86]	0.00	0.00[-0.43,0.43]	1.00	0.07[-0.21,0.35]	0.63

Polymorphism	Ethnicity								Medicine							
	**East Asian**		**Caucasian**		**Indian**		**Russian**		**Risperdone**		**Olanzapine**		**Clozapine**		**Haloperidol**	
	**SMD[95%CI]**	***P* value**	**SMD[95%CI]**	***P* value**	**SMD[95%CI]**	***P* value**	**SMD[95%CI]**	***P* value**	**SMD[95%CI]**	***P* value**	**SMD[95%CI]**	***P* value**	**SMD[95%CI]**	***P* value**	**SMD[95%CI]**	***P* value**
**TT/TC**	-0.28[-0.70,0.13]	0.18	0.74[-0.10,1.59]	0.08	-0.46[-0.73,-0.19]	0.00	0.02[-0.26,0.30]	0.90	-0.03[-0.44,0.37]	0.88	0.86[-0.45,2.16]	0.20	-0.69[-0.98,-0.40]	0.00	0.02[-0.26,0.30]	0.90
**TT/CC**	0.09[-0.24,0.42]	0.58	0.69[-0.10,1.48]	0.09	-0.28[-0.55,-0.02]	0.04	0.02[-0.26,0.30]	0.89	0.06[-0.28,0.41]	0.71	0.84[-0.40,2.09]	0.18	0.06[-0.22,0.34]	0.66	0.02[-0.26,0.30]	0.89
**TC/CC**	0.34[0.02,0.65]	0.04	-0.03[-0.54,0.49]	0.92	0.16[-0.11,0.42]	0.25	0.00[-0.28,0.28]	0.98	0.11[-0.12,0.34]	0.33	0.01[-0.77,0.79]	0.98	0.58[0.29,0.86]	0.00	0.00[-0.28,0.28]	0.98

*CI* confidence interval, *SMD* standardized mean difference value of the scale score reduction rate. Differences were considered statistically significant when P ≤ 0.05

Time subgroup analysis showed that the association between the TT > TC polymorphism and scale score reduction rate in 1995–2000 and 2001–2010 subgroups was statistically significant (*P* value were both 0.00), but the comprehensive SMD = -0.69 in the 1995–2000 subgroup, and SMD = 2.71 in the 2001–2010 population. In addition, the TC > CC polymorphism in the 1995–2000 subgroup and TT > CC polymorphism in the 2001–2010 subgroup were also associated with scale score reduction rate to a certain extent, and the SMD value were 0.58 and 2.71, respectively. These results indicated that the scale score reduction rate of patients with the TC genotype in 1995–2000 was higher than that of patients with the TT and CC genotypes, whereas the reduction rate of patients with the TT genotype in 2001–2010 was higher than that of patients with the TC and CC genotypes.

The association of the TT > TC and TT > CC polymorphisms with the scale score reduction rate in the Indian population (ethnicity subgroup analysis) was statistically significant, and the comprehensive SMD values were -0.46 and -0.28, respectively. This finding indicates that the drug efficacy in patients with the TC and CC genotypes was higher than that in patients with the TT genotype. The TC > CC polymorphism in the East Asian population was associated with scale score reduction rate, SMD = 0.34, indicating high reduction rate in patients with the TC genotype. The presence of the TT > TC and TC > CC polymorphisms in the clozapine treatment group affected the scale score reduction rate, and the comprehensive SMD values were -0.69 and 0.58, respectively. This indicated that the scale score reduction rate in patients with the TC genotype was higher than that in patients with the TT and TC genotypes.

#### Publication bias

**Fig. 2:**
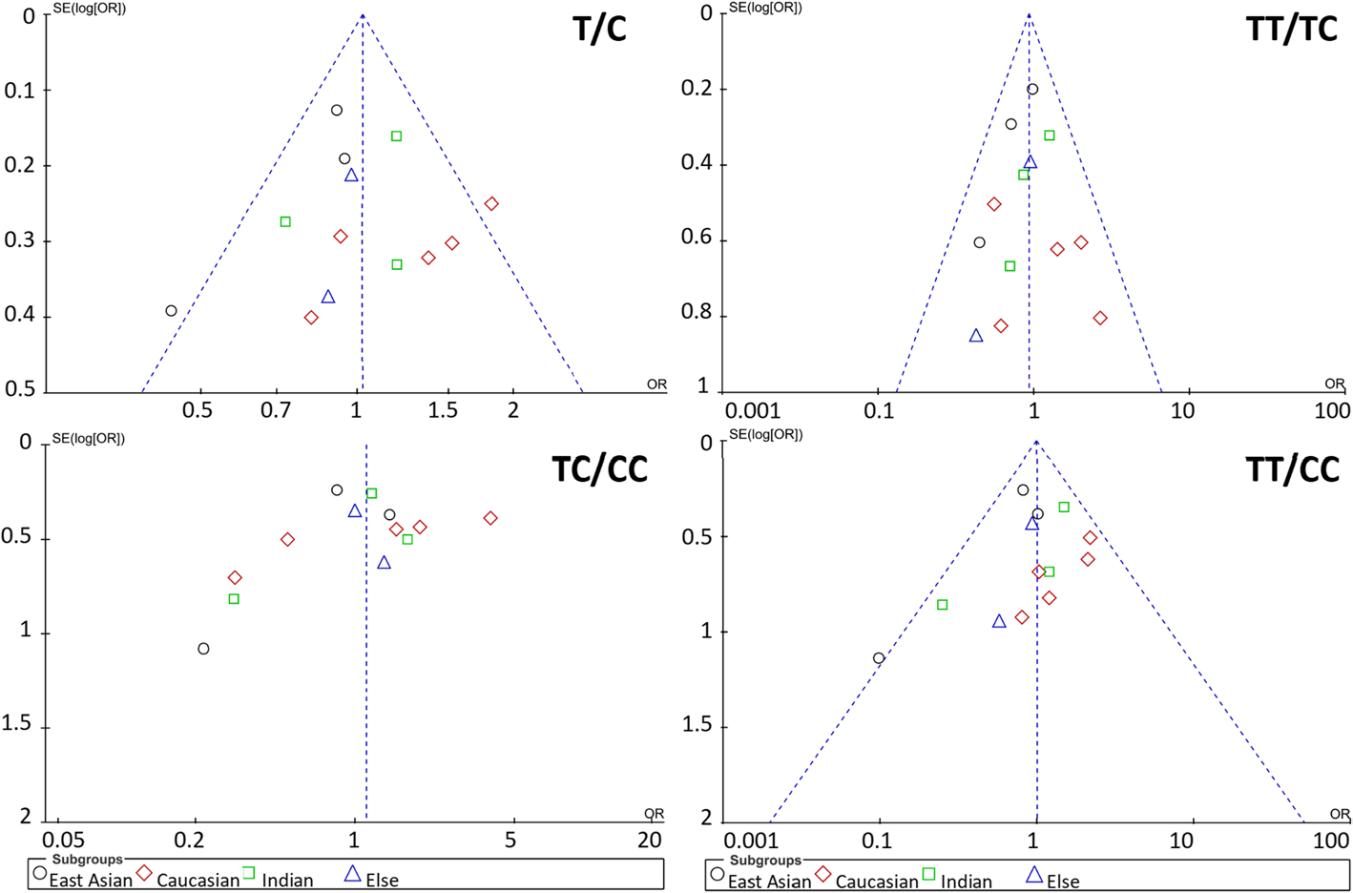
Funnel plot of publication bias analysis for studies with responder rate

**Fig. 3:**
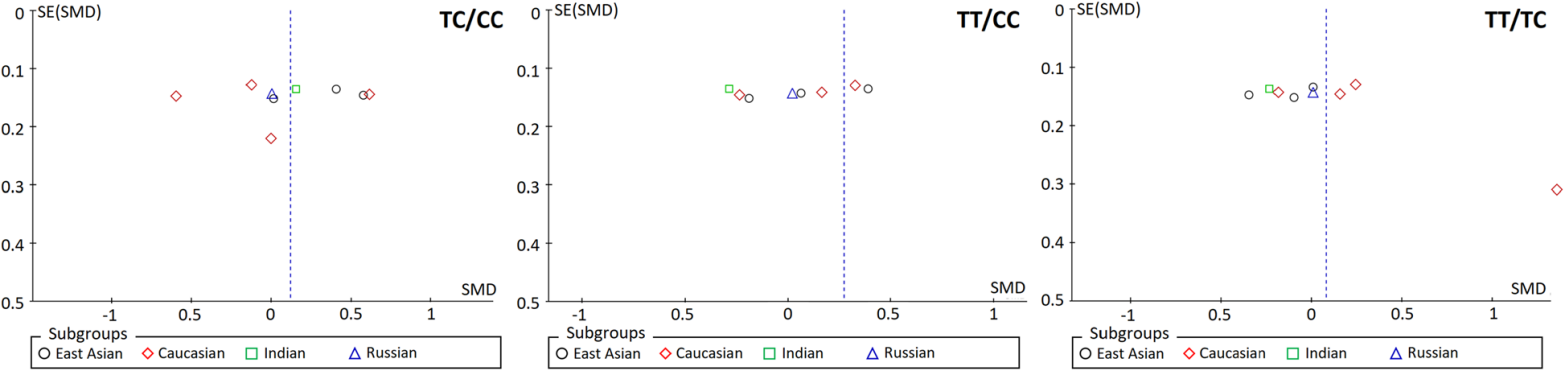
Funnel plots of publication bias analysis for studies with scale score reduction rate

Next, the publication bias of the responder rate and scale score reduction rate related study was evaluated. For the responder rate, the funnel plot was plotted with the OR value as horizontal coordinate and the standard error of log (OR) [SE(log (OR))] value as vertical coordinate. The funnel plot of the meta-analysis of the correlation between alleles and three genotypes and the responder rate was symmetrical (Fig. 2). For the scale score reduction rate, the funnel plot was plotted with SMD value as horizontal coordinate and standard error of SMD [SE(SMD)] value as vertical coordinate. The funnel plot of the meta-analysis of the correlation between the three genotypes and scale score reduction rate also showed high symmetry (Fig. 3). Therefore, no significant publication bias was noted on the studies meta-analysed in this paper.

#### Sensitivity analysis

**Fig. 4:**
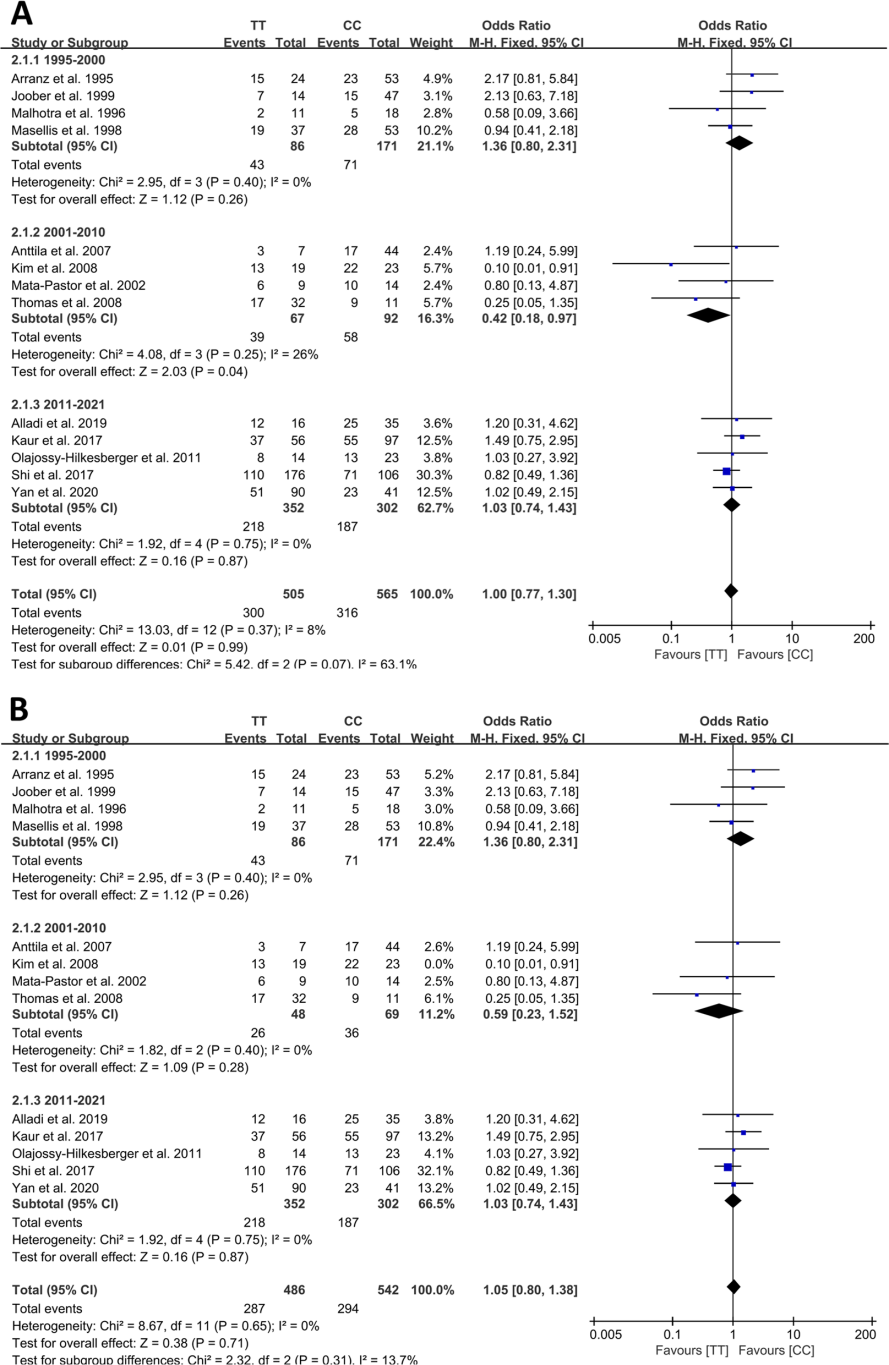
Forest plot of the effect of the TT > CC polymorphism on antipsychotic drug responder rate. **A** Included Kim et al.; (**B**) Excluded Kim et al.

To evaluate the impact of specific studies on the stability of OR and SMD. The sensitivity analysis was carried out by one-by-one elimination method. Excluding studies one by one had no effect on the stability of SMD. For OR, excluding the study by Kim et al. [22] in 2001–2010 population, the result was changed that the TT > CC polymorphism was not associated with the pharmacodynamics of response to antipsychotic drugs (Fig. 4).

## Discussion

The rs6313 (102 T > C) polymorphism in the *5-HTR2A* gene has been proposed to be moderately associated with antipsychotic drug efficacy, but the results of previous studies were inconsistent due to differences in time, sample size, ethnicity, drug, efficacy evaluation methods, and gene polymorphism evaluation methods. In this study, 18 studies that reported on the association between the rs6313 polymorphism (allele and genotype) and antipsychotic drug efficacy (responder rate and scale score reduction rate) from 1995 to 2021 were included for the meta-analysis, and further subgroup analysis was conducted based on time, drug, and ethnicity.

Meta-analysis of the responder data showed that the rs6313 allele T > C polymorphism was not associated with the antipsychotic drug responder rate, but in the ethnicity subgroup analysis, higher responder rate was noted in Caucasian patients with the T allele, so ethnicity differences may influence the efficacy of drug therapy. Further, the analysis of the TT > CC polymorphism showed treatment efficacy was higher in the patients with the CC genotype in 2001–2010 (Fig. 4A). Among the included studies, only Kim et al. [23] used GGI score < 4 as the standard to determine responders to drug treatment. We believe that the criteria of that study were quite different from those of other studies, which led to apparently better efficacy in patients with the CC genotype. After the study by Kim et al. [23] was excluded, the TT > CC polymorphism was not associated with the pharmacodynamics of response to antipsychotic drugs (Fig. 4B).

Meta-analysis of the scale score reduction rate data showed that the TT > TC, TT > CC, and TC > CC polymorphisms were not associated with antipsychotic drug efficacy. Time subgroup analysis showed that in relation to the TT > TC variation, patients with the TT genotype in the 1995–2000 population had better drug treatment efficacy, whereas in the 2001–2010 population, drug treatment produced stronger effect in patients with the TC genotype, that is, the conclusions in the two populations were opposite. We speculate that this was due to the small sample sizes of 97 and 41 individuals, respectively, which were included in only one study for both groups (Table 3). Similarly, the association between the TT > CC and TC > CC polymorphisms and scale score reduction rate in the 1995–2000 and 2001–2010 populations also needs to be further verified by expanding the sample size. Ethnicity subgroup analysis showed that both TT > TC and TT > CC polymorphisms were associated with the drug scale score reduction rate in the Indian population (*P* < 0.05, SMD < 0). Therefore, Indian patients with the TT genotype had the lowest score reduction rate and were poor drug treatment responders. However, only one study with a sample size of 109 was performed in the Indian population. More studies are needed to confirm this conclusion. In the Caucasian population, however, the opposite was true, as higher rate of responders was observed in patients with the T allele. In the east Asian population, the TC > CC polymorphism was related to the scale score reduction rate, which was higher in the patients with the TC genotype than in those with the CC genotype. In other words, drug treatment effect was stronger in the patients with the TC genotype and weaker in those with the CC genotype. Drug subgroup analysis showed that patients with the TC genotype had a higher score reduction rate than those with the TT and CC genotype after clozapine treatment. Subgroup analysis of the responder rate also showed that the therapeutic effect of clozapine in patients with the TC genotype was superior to that achieved in patients with the CC genotype. Therefore, in the clozapine-treated population, patients with the TC genotype benefited from treatment more than patients with the CC genotype.

Therefore, antipsychotic effects were affected by drug, time of study, and ethnicity. That may be due to the distinct mechanisms or sites of action of different drugs, variable ethnic genetic backgrounds, and the influence of the publication time factor could be explained by the progress of experimental technology in the past decade. More studies should be performed to reduce the influence of experimental techniques on the results. This study showed that in the east Asian population and clozapine-treated population, drug efficacy is higher in patients with the heterozygous TC genotype. It has been speculated that the diversity of protein structural units might lead to the change of regulatory functions, which could be beneficial for the drug effects, but the specific mechanism of such interaction will need to be further studied. The C allele of T102 may lead to decreased promoter activity in certain brain regions and decreased 5-HT2A receptor density, leading to the lower drug efficacy [30]. In addition, besides *5-HTR2A*, a variety of enzyme-encoding genes are involved in the metabolism of antipsychotic drugs [31]. Therefore, the association between different gene polymorphisms and antipsychotics needs to be comprehensively analysed, and effects of polymorphisms in genes encoding various enzymes on drug efficacy need to be studied in greater detail.

## Conclusion

The association between the rs6313 polymorphism in the *5-HTR2A* gene and antipsychotic drug efficacy is mainly affected by drug and ethnicity factors. Patients with the T allele fared better in the Caucasian population, whereas the opposite was true in the Indian population. Further, patients with the TC genotype fared better in the east Asian population. Meanwhile, clozapine was effective in patients with the TC genotype.

## Data Availability

All data generated and/or analyzed during this study are included in this published article.
